# Portable Thiocholine-Based Sensor for Monitoring Blood Cholinesterase Activity and Detecting Organophosphate and Carbamate Pesticides Using Personal Glucose Meters

**DOI:** 10.3390/foods14071136

**Published:** 2025-03-25

**Authors:** Udomsap Jaitham, Tipsuda Pintakham, Nan Ei Moh Moh Kyi, Muhammad Samar, Peerapong Jeeno, Surat Hongsibsong, Supansa Pata, Anurak Wongta

**Affiliations:** 1 School of Health Sciences Research, Research Institute for Health Sciences, Chiang Mai University, Chiang Mai 50200, Thailand; udomsap_j@cmu.ac.th (U.J.); pintakham.fah13@gmail.com (T.P.); naneimohmoh_kyi@cmu.ac.th (N.E.M.M.K.); muhammad_samar@cmu.ac.th (M.S.); peerapong_jeen@cmu.ac.th (P.J.); surat.hongsibsong@cmu.ac.th (S.H.); 2 Environmental and Occupational Health Sciences Unit, Research Institute for Health Sciences, Chiang Mai University, Chiang Mai 50200, Thailand; 3 Department of Medical Technology, Faculty of Associate Medical Science, Chiang Mai University, Chiang Mai 50200, Thailand; supansa.pata@cmu.ac.th

**Keywords:** cholinesterase activity sensor, pesticide sensor, personal glucose meter, organophosphorus, carbamate detection, thiocholine

## Abstract

The widespread use of organophosphate and carbamate pesticides in agriculture poses significant health risks due to their cholinesterase (ChE) inhibitory effects. However, existing detection methods are often expensive and require specialized facilities, limiting their accessibility. This study developed a cost-effective, portable, and sensitive sensor using personal glucose meter (PGM) technology to detect ChE activity in human blood and pesticide residues in vegetables. A thiocholine-based assay was designed to measure ChE activity via PGM, enabling the assessment of enzyme inhibition caused by pesticide exposure. The optimized PGM-based sensor achieved limits of detection (LODs) of 0.138 ppm for mevinphos and 0.113 ppm for carbofuran in standard solutions, with strong correlations (R > 0.99) between standard and fortified samples, indicating high sensitivity and accuracy. The method demonstrated reliable detection of ChE inhibition at pesticide concentrations as low as 0.05 ppm. The developed sensor offers a portable and efficient tool for point-of-care diagnostics, environmental monitoring, and food safety applications. This approach enhances public health protection by enabling accessible pesticide detection. Future work will focus on expanding detection capabilities, improving specificity and stability, and conducting clinical validation for broader applications.

## 1. Introduction

Natural and synthetic pesticides are utilized to address agricultural challenges [1]. Their classification hinges on chemical structure, action mechanism, risk potential, and application techniques [2]. The rise in commercial agriculture since the mid-20th century has greatly augmented pesticide utilization, enhancing crop yields [3]. On the other hand, the inadequate regulation in the application of pesticides has culminated in the pollution of soil, water, and air, thereby escalating issues tied to environmental health and food safety [4]. Residues of pesticides permeate the food ecosystem through polluted agricultural goods, processed meals, water, and earth, threatening well-being, particularly among those working on farms [5]. Organophosphates (OPs) and carbamates (CMs) are prevalent due to their effectiveness and extensive applicability. In the Asia–Pacific region, OPs comprise around 40% of global pesticide utilization [6]. Likewise, CMs, classified as cholinesterase inhibitors, are commonly used for pest management [7]. Both OPs and CMs inhibit acetylcholinesterase (AChE), a key enzyme in neural signaling, leading to severe health effects, including neurological disorders, respiratory failure, and potentially fatal outcomes. The measurement of cholinesterase (ChE) in blood is a recognized technique to assess exposure to OPs and CMs, serving as an indicator of their harmful effects in both human and animal groups [8,9,10,11]. Given the prevalent application of these pesticides and their health implications, it is critical to develop rapid, precise, and portable detection techniques for monitoring cholinesterase inhibition and pesticide residues in both environmental and clinical contexts [12,13].

The conventional analytical techniques for the detection of pesticides are gas chromatography (GC), liquid chromatography (LC), high-performance liquid chromatography (HPLC), gas chromatography–mass spectrometry (GC–MS), liquid chromatography–mass spectrometry (LC-MS), micellar electrokinetic capillary chromatography, and capillary electrophoresis (CE) [14,15,16]. Despite the increased specificity, selectivity, and sensitivity that these analytical techniques provide, they are challenged by high costs, extended durations for analysis, and the necessity for specialized machinery and knowledgeable operators. Recent innovations, including electrochemical biosensors and fluorescence sensors, present portable alternatives; however, their intricacy and cost inhibit extensive implementation [17]. Probes and sensors that are cholinesterase-activatable and constructed from nanomaterials have revealed impressive sensitivity in pinpointing OPs and CMs. Nonetheless, their practical application in field environments and resource-limited settings remains constrained [18,19].

Electrochemical glucose biosensors have improved through nanomaterial incorporation, enhancing key performance metrics. Non-enzymatic glucose biosensors utilize nanomaterials to augment electrical and catalytic efficiency, ensuring increased reliability [20]. A viable option is the first-generation amperometric glucose biosensor, featuring a Prussian Blue-modified electrode with a scaffold derived from vegetable waste, promoting efficiency and sustainability [21]. These innovations underscore the adaptability of electrochemical biosensors, applicable to environmental and food safety sectors. Screen-printed electrodes (SPEs) are prevalent due to their economic viability, flexibility, and effectiveness in point-of-care diagnostics. The screen-printing technology facilitates mass production and tailoring, rendering them optimal for various applications [22,23].

Personal blood glucose meters (PGMs) are a cost-effective and accessible biosensing platform for diabetes management [24]. Their widespread availability allows for potential applications such as environmental and biomedical sensors. Initially designed for glucose detection, PGMs had limited applicability in rapid analysis contexts. However, Lu et al. [25] advanced their functionality by integrating glucose detection with target assessment via functionalized DNA-coupled glucose invertase. This innovation enabled the quantification of non-sugar components based on glucose levels, thus overcoming previous limitations. Consequently, PGMs have evolved to detect various molecules beyond sugars. Recent research has investigated electrochemical processes initiated by thiocholine for identifying pesticide residues and assessing cholinesterase inhibition [24,26,27].

Thiocholine (TCh) electrochemical detection has emerged as an effective method for monitoring OP exposure and assessing ChE activity. OPs inhibit AChE, reducing TCh production and enabling the direct quantification of OP residues. Recent advancements in portable AChE-based electrochemical sensors, particularly non-material-modified screen-printed carbon electrode (SPCE) sensors, have significantly enhanced the detection sensitivity of various OPs, including chlorpyrifos and isocarbofos [28,29]. Additionally, innovative sensing techniques with femto-molar sensitivity present new opportunities for environmental monitoring [30]. Ferricyanide ([Fe(CN)_6_]^3−^), commonly used as a mediator in glucose test strips, also plays a role in synthetic peptide applications by converting bis(thiols) into disulfides [31]. Similarly, the electrochemical oxidation of sulfhydryl groups in TCh leads to disulfide formation, generating an electron transfer signal quantifiable via electrochemical detection [32,33]. Given these properties, it is hypothesized that [Fe(CN)_6_]^3−^ may facilitate TCh oxidation to its disulfide form, enabling direct electrochemical detection. If validated, this mechanism would allow personal glucose meters (PGMs) to measure TCh levels as easily as blood glucose, providing a rapid, accessible, and straightforward method for detecting AChE inhibition and OP exposure.

Given the limitations of conventional pesticide detection methods such as GC-MS, LC-MS, and ELISA, which require specialized equipment and training, PGMs are widely available, affordable, and user-friendly. The PGM-based method is an alternative way to use in the field. The aim of this study is to develop a sensitive, portable, and reasonably priced sensor that can detect cholinesterase inhibition and OP and CM pesticide residues. Our proposal specifically calls for the development and validation of thiocholine-based sensors using PGMs, allowing for the real-time monitoring of human pesticide exposure and environmental pollution. By integrating these technologies, we intend to expand their use in clinical and agricultural settings, thereby enhancing public health monitoring and worker safety.

## 2. Materials and Methods

### 2.1. Reagents and Apparatus

Cricket cholinesterase (ChE) was obtained from our previous study [34]. Acetylthiocholine iodide (ATCh) and 5,5′-dithiobis (2-nitrobenzoic acid) (DTNB) were purchased from Sigma Aldrich (St. Louis, MO 63178, USA). Standard pesticides, including mevinphos and carbofuran, were procured from Dr. Ehrenstorfer GmbH (Augsburg, Germany). The personal glucose meter (PGM, Sannuo; Safe AQ smart, detection range: 20–600 mg/dL) was purchased from Changsha Sinocare Inc. (Changsha, China).

### 2.2. Preparation of Solutions

#### 2.2.1. Acetylthiocholine Solution

A 15 mM acetylthiocholine iodide solution was prepared by dissolving 4.34 mg of ATCh (MW: 289.18 g/mol) in 1 mL of distilled water. The solution was mixed using a vortex mixer until complete dissolution and stored at −20 °C to prevent degradation.

#### 2.2.2. Phosphate-Buffer Solution (PBS)

PBS (pH 7.4) was prepared with 137 mmol/L NaCl, 2.7 mmol/L KCl, 10 mmol/L Na_2_HPO_3_, and 1.8 mmol/L KH_2_PO_4_, yielding a phosphate concentration of 11.8 mmol/L.

#### 2.2.3. Specimen Preparation

Blood samples were collected via finger prick from late October to late December 2023. A total of 73 samples, each approximately 150 µL, were collected using heparinized capillary tubes. The blood was subsequently transferred into centrifuge tubes. From each sample, 50 µL was pooled to create a control blood sample for method development. The samples were then transported in an ice-cooled container to the Environmental and Occupational Health Science Laboratory at Chiang Mai University and stored at −20 °C until further analysis.

### 2.3. Blood Cholinesterase Analysis Using Corrected PGM Readout

**Figure 1:**
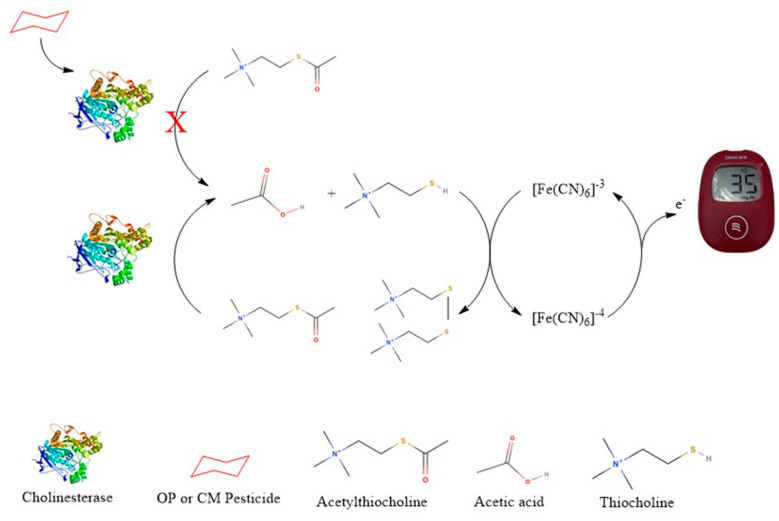
Schematic diagram of the thiocholine-based sensor for detecting cholinesterase activity and pesticide-induced inhibition; Letter X means inhibit.

The PGM-based biosensor detects thiocholine, the enzymatic product of acetylthiocholine (ATCh), to assess cholinesterase activity. Cholinesterase (from crickets or whole blood) hydrolyzes ATCh, producing thiocholine, which is applied to a modified PGM test strip for electrochemical oxidation. The PGM quantifies the resulting current, where a lower signal indicates enzyme inhibition due to pesticide exposure. This method offers a rapid, portable, and cost-effective alternative for pesticide detection as shown in the schematic diagram below (Figure 1).

#### 2.3.1. Optimization of Whole Blood ChE Activity Measurement

The method for evaluating thiocholine-related signals using corrected PGM readouts was adapted from a previous study [27]. The effects of ATCh concentration, incubation time, and pH were optimized using whole blood samples from our prior research.

Whole blood samples were diluted 1:10 by mixing 100 µL of whole blood with 900 µL PBS (pH 8.0). Sample blanks were prepared by mixing 50 µL of the diluted blood sample with 150 µL PBS, while reagent blanks contained 175 µL PBS and 25 µL of ATCh at 5, 10, or 15 mM concentrations. Test reactions were conducted by adding 50 µL of the sample to 125 µL PBS and 25 µL of ATCh, incubated at 25 °C, with PGM readings recorded after 5, 10, and 15 min.

The corrected PGM readout was calculated using:

(1) Corrected PGM readout (mg/dL)=Measured result−Sample blank−Reagent blank

where

Measured result: PGM reading from the reaction tube (blood sample, PBS, and ATCh) after 10 min of incubation.
Sample blank: PGM reading from the tube containing blood sample and PBS (no ATCh).
Reagent blank: PGM reading from the tube containing PBS and ATCh (no blood sample).

#### 2.3.2. Dose–Response Analysis of OP and CM Pesticides Using Corrected PGM Readouts

To determine the effect of OPs and CMs on thiocholine signals, blood samples were diluted 1:10 in PBS (pH 8.0). Test samples were prepared by mixing 50 µL of diluted blood with 50 µL of 1 ppm mevinphos, incubated for 5–30 min to optimize inhibition time. Control samples contained blood without mevinphos. Following inhibition, 25 µL of 15 mM ATCh and 75 µL PBS were added, and incubated for 10 min at 25 °C, and PGM readings were recorded.

The percentage of cholinesterase inhibition was calculated as: [35]

(2) $$\%Inhibition=\frac{\left(Corrected\;PGM\;control-Corrected\;PGM\;inhibited\right)}{\left(Corrected\;PGM\;control\right)}\times 100$$

where

Corrected PGM control: PGM readout without pesticide exposure.
Corrected PGM inhibited: PGM readout with pesticide exposure.

For dose–response analysis, blood samples were mixed with 0.05–1 ppm of mevinphos or carbofuran and incubated for 30 min before measuring corrected PGM readouts and inhibition percentages.

### 2.4. Detection of OP and CM Residues Using Cricket ChE and Corrected PGM Readout

#### 2.4.1. Optimization of Cricket ChE Activity Measurement

The effects of ATCh concentration and incubation time on cricket ChE activity were evaluated. Cricket ChE samples were diluted 1:10 in PBS (pH 8.0). Blanks and reagent controls were prepared as previously described. Test reactions of each ATCh concentration (5, 10, and 15 mM) were conducted at 25 °C for 5, 10, 15 and 20 min. The corrected PGM readouts were recorded. The ATCh concentration that resulted in the highest enzyme activity was selected for subsequent experiments.

Then, the test was conducted to determine the optimal incubation time for cricket ChE activity measurement. ChE activity was assessed using 15 mM ATCh at incubation times of 5, 10, 15, and 20 min at 25 °C. The incubation time that resulted in the highest enzyme activity was selected as the optimal condition for the PGM readout method.

A Michaelis-Menten graph was constructed to determine the Michaelis constant (Km) and maximum velocity (Vmax). The Michaelis-Menten model, a fundamental concept in enzymology, describes the relationship between reaction rate (v) and substrate concentration ([S]) [34], expressed as:

v = Vmax[S]/(Km + [S])

where

v = reaction rate
Vmax = maximum reaction rate at saturating substrate levels
[S] = substrate concentration
Km = substrate concentration at half of Vmax, indicating enzyme affinity

The reproducibility of the PGM readout method was evaluated through intra-assay and inter-assay variability analysis. For intra-assay variability, the same sample was tested in ten tests within a single experimental run. The inter-assay variability was assessed six times by analyzing the same sample across two independent experiments conducted on different days. The standard deviation (SD) and coefficient of variation (CV) were calculated for both intra- and inter-assay assessments.

#### 2.4.2. Pesticide Inhibition Analysis Using Cricket ChE

To optimize inhibition conditions, diluted cricket ChE samples were incubated with 1 ppm mevinphos for 5, 10, 20, 30, and 40 min. Following inhibition, 25 µL of 15 mM ATCh was added, and enzyme activity was measured using the corrected PGM readout method. Percentage inhibition was calculated as previously described. The incubation time that resulted in the highest enzyme inhibition was selected for subsequent experiments.

To assess dose–response effects, cricket ChE was exposed to 0.008–1 ppm mevinphos or carbofuran, incubated for 30 min, and tested using the corrected PGM method. The IC50 and IC20 values were determined using GraphPad Prism (v4.01), with IC20 serving as the method’s limit of detection (LOD) [36].

#### 2.4.3. Sample Preparation for Pesticide Detection

##### Effect of Methanol on PGM Readouts

To evaluate methanol (MeOH) interference, test solutions were prepared with 1–5% MeOH in PBS (pH 8.0). Samples containing cricket ChE, ATCh, and MeOH solutions were incubated for 10 min at 25 °C, and corrected PGM readouts were analyzed.

##### Sample Extraction Procedure

Pesticide-free cabbage (confirmed by GC-FPD analysis) [37] was used as a test matrix. For extraction, 2 g of finely chopped cabbage was shaken with 4 mL of dichloromethane for 5 min. A 2 mL aliquot of the extract was transferred to a glass tube and evaporated to dryness in a hot water bath. The residue was reconstituted in 200 µL of dichloromethane, followed by 200 µL of 5% methanol in PBS (pH 8.0), and thoroughly mixed. Dichloromethane was removed by heating at 60 °C in a hot water bath, and the resulting solution was used for pesticide analysis.

#### 2.4.4. Pesticide Detection in Fortified Samples

Pesticide-free cabbage samples spiked with mevinphos and carbofuran at concentrations ranging from 0.008 to 1 ppm were used to evaluate the dose–response relationship. Following extraction, 50 µL of the extract was mixed with 50 µL of diluted cricket ChE (1:10 in PBS, pH 8.0) and incubated for 30 min. The reaction mixture was supplemented with 25 µL of 15 mM ATCh and 75 µL of PBS to a final volume of 200 µL. After a 10 min incubation at 25 °C, corrected PGM readouts were obtained, and the percentage inhibition was calculated using the previously described formula. The IC50 and IC20 values were determined as in our previous study [34], with IC20 being used as the LOD for the fortified sample.

The sensor’s accuracy for pesticide residue detection was evaluated through a recovery study using spiked cabbage samples. Pesticide-free samples were fortified with known concentrations of mevinphos and carbofuran, extracted using the established protocol, and analyzed against standard solutions. Recovery rates were calculated as follows:

$$Recovery\;\left(\%\right)=\frac{\left(Inhibition\;of\;standard\;pesticide\right)}{\left(Inhibition\;of\;spiked\;sample\right)}\times 100$$

### 2.5. Statistical Analysis

The test was performed in duplicate. All corrected PGM readouts were reported as mean ± standard deviation. Inhibition curves and IC50 values were determined using GraphPad Prism (v4.01). Differences in ChE activity were analyzed using *t*-tests and one-way ANOVA, while Pearson correlation was used to assess the relationship between standard and fortified pesticide samples. A *p*-value < 0.05 was considered statistically significant.

## 3. Results

### 3.1. Blood Cholinesterase Activity

#### 3.1.1. The Effect of ATCh Concentration and Incubation Time on Blood ChE Activity

**Figure 2:**
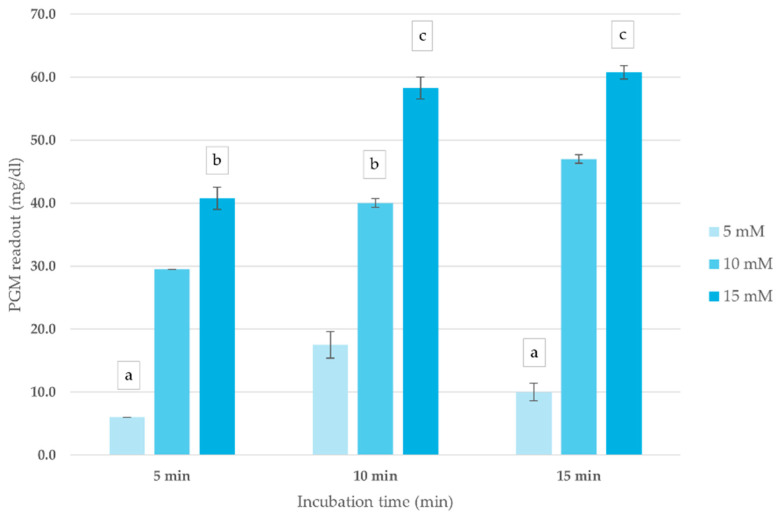
Effect of ATCh concentration and incubation time on ChE activity (mg/dL) in whole blood sample. PGM readout was measured at different ATCh concentrations (5, 10, and 15 mM) at 25 °C over 15 min. Means followed by the same letter are not significantly different (*p* > 0.05; one-way ANOVA; post hoc Bonferroni Multiple Comparisons).

The impact of ATCh concentration and incubation time on blood ChE activity was assessed to optimize conditions for the corrected PGM readout method. As shown in Figure 2, PGM readouts exhibited a concentration-dependent response, with the highest signal intensity recorded at 15 mM ATCh across all incubation periods. Increasing incubation time up to 10 min enhanced PGM readout, after which the signal plateaued, indicating reaction saturation. Based on these findings, an ATCh concentration of 15 mM and a 10 min incubation time were selected for subsequent analyses.

#### 3.1.2. Optimal Incubation Time for Pesticide-Induced Blood ChE Activity Inhibition

**Figure 3:**
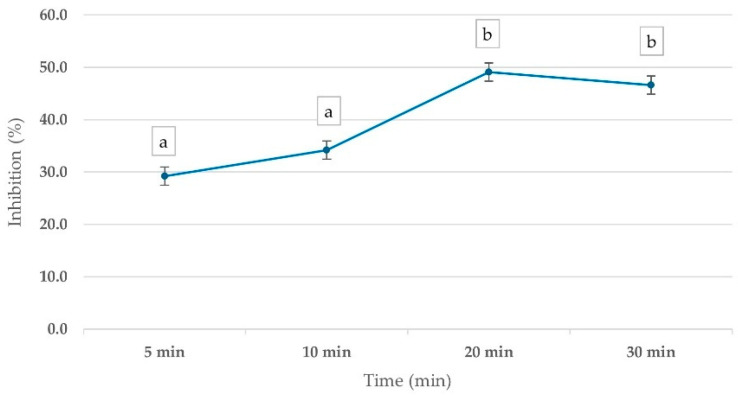
Percentage inhibition of ChE activity in whole blood samples, measured by corrected PGM readout, using 15 mM ATCh substrate after 10 min reaction time. Whole blood was incubated with 1 ppm Mevinphos for 5–30 min before measurement. Means followed by the same letter are not significantly different (*p* > 0.05; one-way ANOVA; post hoc Bonferroni Multiple Comparisons).

To evaluate the optimal time for pesticide inhibitory effects on ChE activity, whole blood samples were incubated with 1 ppm mevinphos for various time intervals (5–30 min). The results demonstrated a time-dependent inhibition of ChE activity, with PGM readouts showing a 29.2% inhibition at 5 min, increasing to 49.1% at 20 min before stabilizing. This trend suggests that 20 min is the optimal incubation time for pesticide inhibition assessment (Figure 3).

#### 3.1.3. Effect of OP and CM Pesticide Concentrations on Whole Blood ChE Activity

The effect of increasing mevinphos and carbofuran concentrations on ChE activity was investigated. As shown in Table 1, both pesticides exhibited a dose-dependent inhibitory effect, with mevinphos demonstrating stronger inhibition. At 1 ppm, mevinphos inhibited 53.1% of ChE activity, while carbofuran caused a 44.9% reduction. At lower concentrations (0.05–0.5 ppm), mevinphos consistently exhibited higher inhibitory potency than carbofuran. Furthermore, we observed a significantly decrease in values between spiking pesticides and baseline at all concentrations (0.05–1 ppm). The inhibition data were analyzed using an independent *t*-test, comparing pesticide-exposed samples with control (pesticide-free) samples. The results showed a statistically significant difference (*p* < 0.05) between groups, confirming that pesticide presence led to a measurable reduction in cholinesterase activity.

### 3.2. Result of OP and CM Residue Detection Using Cricket ChE

#### 3.2.1. Effects of ATCh Concentration and Incubation Time on Cricket ChE Activity

**Figure 4:**
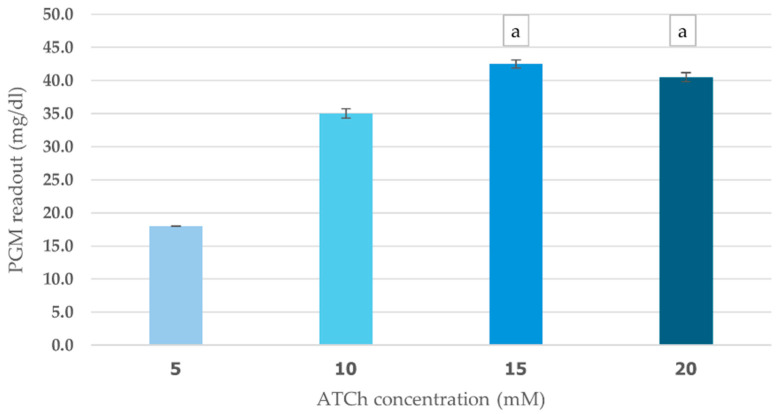
Effect of ATCh concentration on cricket ChE activity. ChE activity was measured at 25 °C for 10 min using the PGM readout method with ATCh concentrations ranging from 5 to 20 mM. Means followed by the same letter are not significantly different (*p* > 0.05; one-way ANOVA; post hoc Bonferroni Multiple Comparisons).

The effects of ATCh concentration (5–20 mM) and incubation time (5–20 min) on cricket ChE performance were evaluated using PGM readout method. This study found a clear positive relationship between ATCh concentration and enzyme activity. As ATCh levels increased from 5 to 15 mM, PGM values increased significantly, peaking at 42.5 mg/dL (Figure 4). This suggests that a greater availability of substrate boosts enzyme activity. However, at 20 mM ATCh, PGM values slightly declined to 40.5 mg/dL, which could indicate that the enzyme was reaching saturation or experiencing some inhibitory effects at higher concentrations. The enzyme kinetics analysis revealed Km of 6 mM and Vmax of 43 mg/dL for the ATCh substrate (Supplementary File).

**Figure 5:**
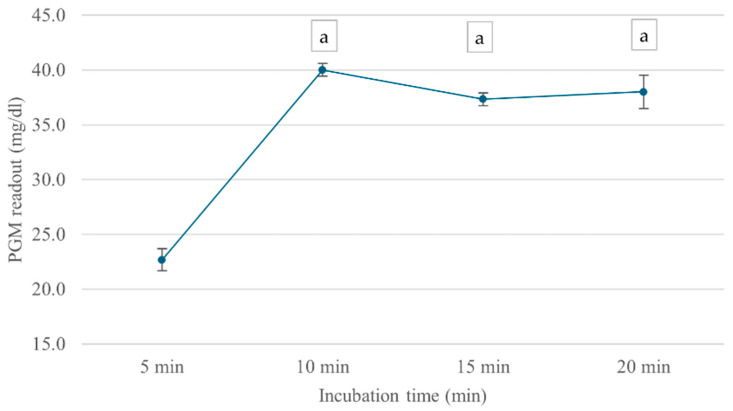
Effect of incubation time on cricket ChE activity. ChE activity was measured at incubation times ranging from 5 to 20 min using the PGM readout method at 25 °C. Means followed by the same letter are not significantly different (*p* > 0.05; one-way ANOVA; post hoc Bonferroni Multiple Comparisons).

The incubation time-dependent analysis showed that ChE activity continually increased over the first 10 min, reaching a maximum PGM value of 40 mg/dL, as shown in Figure 5. However, at 15 min, ChE activity slightly declined, possibly due to enzyme instability or product inhibition over extended incubation periods. Based on these findings, an ATCh concentration of 15 mM and a 10 min incubation time were selected for subsequent analyses.

#### 3.2.2. Reproducibility and Variability Analysis

The intra-assay variability of the PGM-based sensor was determined with an SD of 1.77 mg/dL and a CV% of 4.24%, indicating high precision within a single experimental run. The inter-assay variability was assessed across multiple independent tests, yielding an SD of 1.6 mg/dL and a CV% of 3.7%, demonstrating consistent performance between different experiments (Supplementary File). These findings suggest that the developed biosensor provides reliable and reproducible results for cholinesterase inhibition detection.

#### 3.2.3. Optimal Incubation Time for Pesticide-Induced Cricket ChE Activity Inhibition

**Figure 6:**
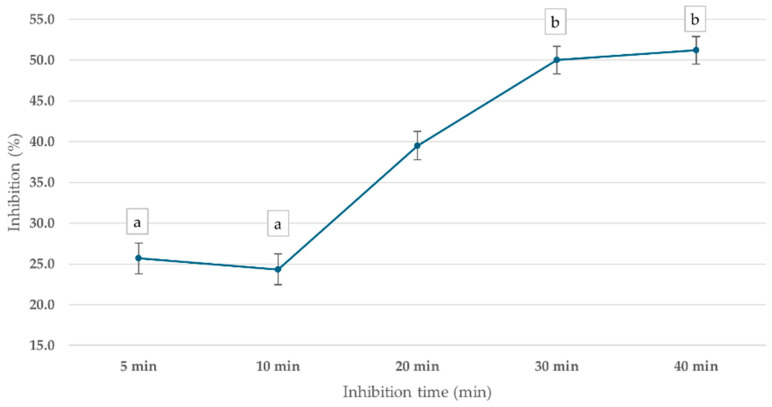
Effect of inhibition time on cricket ChE activity using the PGM readout method. Cricket ChE samples were incubated with 1 ppm mevinphos for 5–40 min, followed by measurement with 15 mM ATCh at 25 °C. Means followed by the same letter are not significantly different (*p* > 0.05; one-way ANOVA; post hoc Bonferroni Multiple Comparisons).

ChE inhibition was observed across all time points, with the percentage inhibition increasing over time, as shown in Figure 6. After 5 min of incubation, ChE activity was inhibited by 25.7%. At 10 min, the inhibition slightly decreased to 24.3%, suggesting a temporary fluctuation in enzyme response. However, by 20 min, inhibition returned to 39.5%, and the highest inhibition (50.0%) was observed at 30 min, indicating a time-dependent increase in mevinphos-induced ChE inhibition. The 30 min incubation time was used in further steps.

#### 3.2.4. Effect of Methanol Concentration on Cricket ChE Activity

The impact of MeOH concentration in PBS (pH 8.0) on cricket ChE activity was assessed using PGM readouts at six different MeOH levels (0–5% *v*/*v*). The results, summarized in Table 2, indicate that MeOH concentrations up to 5% did not significantly affect ChE activity (*p* > 0.05). The PGM readouts remained relatively stable, with values ranging from 40.5 to 44.5 mg/dL, demonstrating that the presence of MeOH in PBS had minimal influence on enzymatic activity. The highest PGM readout (44.5 mg/dL) was observed at 1% MeOH, while the lowest (40.5 mg/dL) was recorded at 4% MeOH. These results suggest that MeOH concentrations up to 5% can be used in the assay without significantly impacting ChE activity, ensuring compatibility with sample preparation methods involving MeOH-based extractions.

#### 3.2.5. Limit of Detection and Correlation Analysis of Pesticide Inhibition in Standard and Fortified Samples Using the PGM Readout Method

To evaluate the dose–response effects of standard pesticides and fortified samples, cricket ChE was exposed to mevinphos or carbofuran at concentrations ranging from 0.008 to 1 ppm, incubated for 30 min, and analyzed using the corrected PGM method. The LOD for mevinphos and carbofuran was determined in both standard pesticide solutions and fortified samples using the PGM readout method (Table 3). The LOD for mevinphos was 0.138 ppm in standard solutions and 0.164 ppm in fortified samples, while for carbofuran, it was 0.113 ppm in standard solutions and 0.127 ppm in fortified samples.

A strong correlation (R = 0.996 for mevinphos; R = 0.999 for carbofuran) was observed between standard pesticide inhibition and fortified samples at the same concentration, indicating high agreement between the two matrices. The *p*-values (<0.01) confirm that this correlation is statistically significant, suggesting that the PGM inhibition method is reliable for detecting pesticide residues in fortified food samples. These findings demonstrate their potential for practical applications in pesticide screening and food safety analysis.

#### 3.2.6. Recovery of Pesticide Residues in Spiked Samples

The recovery rates for pesticide residues in fortified cabbage samples were determined to evaluate the accuracy of the developed biosensor. The recovery for mevinphos ranged from 90.8% to 118.9%, while for carbofuran, it ranged from 98.4% to 136.1% (Supplementary File). These values indicate that the sensor provides reliable quantification of pesticide residues, though variations may arise due to matrix effects.

## 4. Discussion

Our results highlight the significant potential of PGMs when combined with thiocholine-based tests as practical tools for real-time pesticide exposure monitoring in various scenarios, ranging from clinical diagnostics to environmental surveillance. Our approach introduces a novel pesticide detection strategy by utilizing PGM technology to measure thiocholine-based cholinesterase inhibition. Our approach is affordable, user-friendly, and adaptable for real-time field monitoring, unlike conventional pesticide detection techniques that often require complex instrumentation and trained personnel. Using cricket cholinesterase as a biorecognition element is also a sustainable and cost-effective way to obtain enzymes, which makes it perfect for testing pesticides on-site in medical and agricultural settings. This innovation has significant implications for public health monitoring and occupational exposure assessment, offering a practical alternative to existing methodologies.

### 4.1. Optimization of Assay Conditions for Thiocholine-Based Detection

Optimizing assay conditions is crucial for the sensitive and accurate detection of ChE activity using PGM. Our study identified 15 mM ATCh with a 10 min incubation as the optimal condition, enabling precise whole blood ChE measurement. This aligns with enzyme kinetics principles, where substrate concentration and incubation time influence reaction rates [38]. A dose-dependent response was observed, with a slight signal decline at 20 mM ATCh, suggesting possible substrate saturation or limitations in the PGM’s linear detection range. Further kinetic studies over a broader substrate concentration range could refine assay performance and better define detection limitations for high-abundance analytes. Previous research [27] on organophosphate detection using PGMs highlighted the importance of substrate concentration and reaction duration. Within the 5–50 mM ATCh range, a 5 min reaction produced detectable TCh, though approaching the PGM detection threshold (1.1 mM). The highest PGM signal was observed at 5 mM ATCh, followed by signal attenuation at higher concentrations, likely due to excessive substrate-induced AChE inhibition. In contrast, our study found that 10 mM ATCh produced the highest signal at 10 min, indicating stable enzyme activity and sufficient TCh production.

Our findings extend previous studies by demonstrating that 15 mM ATCh maintains optimal enzyme kinetics without significant substrate inhibition, improving detection accuracy. While prior studies identified 10 mM ATCh as optimal, our data suggest that a slightly higher concentration (15 mM) enhances reaction efficiency while ensuring effective signal detection. Additionally, the signal decline at 20 mM ATCh supports earlier findings that excess substrate may inhibit enzyme activity.

Mevinphos and carbofuran exhibited clear inhibitory effects on total blood ChE activity, with mevinphos demonstrating stronger inhibition, consistent with their classification as potent ChE inhibitors [39]. The concentration-dependent response of the sensors highlights their potential for pesticide quantification and differentiation based on inhibitory strength. This aligns with previous research showing that organophosphate and carbamate pesticides can be effectively detected and quantified using cholinesterase inhibition-based biosensors [40]. However, pesticide toxicity in vivo involves complex metabolic and detoxification processes that extend beyond ChE inhibition alone [12].

The economic use of cricket ChE presents significant advantages for resource-limited applications. However, when comparing cricket ChE with mammalian ChE, differences in surface selectivity, inhibitor sensitivity, and stability must be considered, despite cricket ChE being widely available and cost-effective [34]. Functional variations in mammalian AChE expressed in different systems may impact biosensor performance, as highlighted by comparative studies [41]. Further research is needed to evaluate biosensing performance across different scenarios. For detecting organophosphate and carbamate pesticides, the development of a micro electrometric technique using cricket ChE has been explored as a rapid and cost-effective alternative to conventional methods [36]. The selection of an appropriate enzyme is critical for optimizing detection efficiency, as emphasized by previous studies [42].

### 4.2. Comparative Analysis of Detection Limits

The achieved LODs for mevinphos (0.138 ppm in standard solutions and 0.164 ppm in fortified cabbage) and carbofuran (0.113 ppm in standard solutions and 0.127 ppm in fortified cabbage) using our PGM-based method demonstrate competitive sensitivity among portable pesticide sensors. Comparable electrochemical sensors typically report LODs in the 0.2–2.35 ppm range [43], while nanomaterial-enhanced sensors can achieve ppb-level detection but with increased complexity [44]. Fluorescence-based sensors offer high sensitivity but require specialized instrumentation [45], whereas spectrophotometric methods, though widely used in laboratories, are less portable and provide comparable LODs [46]. Our results align with a previous study [27], which reported a 0.5 ppm LOD for paraoxon-ethyl using PGMs, further supporting their applicability. While GC-MS and LC-MS remain the gold standard, achieving ppt-level LODs [47,48], our PGM-based sensor provides a cost-effective and reliable screening tool for rapid field detection, particularly in resource-limited settings.

### 4.3. Strengths and Limitations

Our PGM-based biosensor offers a portable, affordable, and user-friendly solution for on-site pesticide detection, leveraging widely available glucose meters for rapid analysis. The integration of cricket-derived ChE enhances cost-effectiveness and sustainability, while the 10 min assay enables timely decision-making. With competitive sensitivity and accuracy for mevinphos and carbofuran detection, it provides a practical alternative to conventional methods. Unlike GC-MS, LC-MS, and ELISA, which offer high specificity but require costly, time-intensive, and lab-dependent processes, our sensor eliminates these barriers by delivering fast, low-cost, and field-deployable results with minimal training. These advantages make it particularly valuable in resource-limited settings, supporting pesticide monitoring in clinical, agricultural, and environmental applications where traditional techniques are impractical.

Despite these advantages, several challenges still remain that must be validated against a wider range of pesticides and potential matrix interferences. While methanol effects were minimal at low concentrations, higher solvent levels and complex sample compositions require further assessment. Clinical validation is also essential to confirm applicability for human health monitoring. Additionally, the long-term stability of the enzyme and sensor system need improvement for real-world deployment. While PGMs offer cost and portability advantages, their sensitivity and dynamic range are more limited compared to laboratory instruments. Batch-to-batch variability in PGM strips also requires quality control measures to ensure consistent performance [49]. Finally, streamlining the corrected PGM readout method would improve user-friendliness in field applications.

### 4.4. Implications and Future Directions

The portable PGM-based sensor presents transformative potential for pesticide exposure monitoring across clinical, environmental, and occupational settings. In healthcare, it could enable rapid, point-of-care diagnosis of pesticide poisoning in emergency rooms, rural clinics, and agricultural fields. For environmental monitoring, it offers on-site screening of pesticide residues in water, soil, and food, facilitating timely interventions for food safety and contamination control. In occupational health, the sensor provides an affordable and practical tool for routine cholinesterase monitoring among agricultural workers and at-risk populations, supporting early exposure detection and preventive measures.

This study utilizes a thiocholine-based biosensor to assess cholinesterase activity, which varies based on enzyme type and substrate selection. While both AChE and BChE are present in whole blood, our findings show no significant difference in PGM readout between whole blood and cricket cholinesterase, indicating comparable enzymatic activity. However, substrate choice may impact thiocholine production and detection sensitivity. Future research should explore butyrylthiocholine (BTCh) as an alternative substrate to potentially enhance detection, particularly for human blood applications. This optimization could improve sensor performance in both clinical diagnostics and environmental pesticide monitoring.

Future research should expand pesticide detection capabilities, enhance sensitivity and stability, and ensure real-world validation. Key advancements include multiplexed detection, assess the sensor’s performance in complex sample matrices, evaluate potential interferences, and validate its applicability for routine pesticide screening, user-friendly interfaces, and strategies to mitigate PGM variability, further optimizing its use for point-of-care and environmental applications.

### 4.5. Potential Interferences and Food Matrix Limitations

The PGM-based biosensor detects thiocholine electrochemical signals, but false positives may arise from heavy metals or electroactive food compounds (e.g., polyphenols or flavonoids) interfering with detection [50,51]. False negatives can result from enzyme degradation, extreme temperatures, or complex food matrices reducing thiocholine production [52].

Food matrix complexity further affects enzyme activity and sensor accuracy due to electroactive compounds, pH variations, and high lipid or protein content, which can alter pesticide solubility and electrochemical responses [53].

Future research should focus on matrix-specific calibration, sample pre-treatment (filtration, dilution, and extraction), and sensor selectivity enhancements. Despite these challenges, the PGM-based biosensor remains a promising tool for rapid, portable pesticide detection, with further optimization improving its use in food safety and environmental monitoring.

## 5. Conclusions

This study advances portable pesticide detection with a thiocholine-based sensor utilizing PGM for rapid, cost-effective, and sensitive detection of ChE inhibition and OP/CM pesticides. The sensor offers competitive sensitivity, affordability, and ease of use, making it a promising alternative to existing methods. While challenges remain such as enhancing specificity, stability, and sensitivity and addressing PGM variability, this work underscores the strong potential of PGM-based sensors for point-of-care diagnostics, environmental monitoring, and food safety. Future work should focus on improving sensor selectivity, optimizing enzyme stability, refining PGM calibration, and exploring alternative substrates like BTCh to enhance detection in human biomonitoring. By enabling widespread, accessible pesticide screening, this innovation contributes to public health and environmental protection in the face of global pesticide exposure risks.

## Figures and Tables

**Table 1:** Inhibition of blood cholinesterase activity by mevinphos and carbofuran at concentrations ranging from 0.05 to 1 ppm compared to baseline activity.

Insecticide Conc. (ppm)	PGM Read Out (mg/dL)	Inhibition (%)
Baseline 0.0	(49 ± 0.7)	
Mevinphos		
1	23.0 ± 0.7 *	53.1 ± 1.4
0.5	27.0 ± 0.7 *	44.9 ± 1.4
0.25	33.0 ± 0.7 *	32.7 ± 1.4
0.05	35.5 ± 0.0 *	27.6 ± 0.0
Carbofuran		
1	27.0 ± 0.7 *	44.9 ± 1.4
0.5	31.0 ± 0.7 *	36.7 ± 1.4
0.25	36.0 ± 0.7 *	26.5 ± 1.4
0.05	38.0 ± 0.7 *	22.4 ± 1.4

Abbreviations: * Significantly different from baseline activity at *p* < 0.05 (*t*-test).

**Table 2:** Effect of methanol concentration in PBS on cricket ChE activity measured using the PGM readout method.

	Concentration of MeOH in PBS (%)					
	0	1	2	3	4	5
PGM readout (mg/dL)	43 ± 1 ^a^	44.5 ± 0.5 ^a^	42.5 ± 0.5 ^a^	42.5 ± 1.5 ^a^	40.5 ± 0.5 ^a^	41.5 ± 0.5 ^a^

Abbreviations: PGM, portable glucose meter; MeOH, methanol; PBS, phosphate-buffered saline. Means followed by the same letter are not significantly different (*p* > 0.05; one-way ANOVA; post hoc Bonferroni multiple comparisons).

**Table 3:** Correlation between detection limits of standard pesticides and fortified samples using the PGM readout method.

Pesticide Name	LOD (ppm)		R	*p*-Value
	Standard	Fortified Sample		
Mevinphos	0.138	0.164	0.996	<0.01 *
Carbofuran	0.113	0.127	0.999	<0.01 *

Abbreviations: LOD: limit of detection; ppm: part per million. *: Significantly different at *p* < 0.01 by Pearson correlation.

## Data Availability

The original contributions presented in the study are included in the article, further inquiries can be directed to the corresponding author.

## Supplementary Materials

The following supporting information can be downloaded at: https://www.mdpi.com/article/10.3390/foods14071136/s1, Figure S1: Km Vmax; Table S1: Intra batch; Table S2: Inter batch; Table S3: Recovery.
